# Towards Accurate and Precise Image-Guided Radiotherapy: Clinical Applications of the MR-Linac

**DOI:** 10.3390/jcm11144044

**Published:** 2022-07-13

**Authors:** James W. Randall, Nikhil Rammohan, Indra J. Das, Poonam Yadav

**Affiliations:** Northwestern Medicine, Department of Radiation Oncology, Chicago, IL 60611, USA; james.randall@nm.org (J.W.R.); nikhil.rammohan@nm.org (N.R.); indra.das@nm.org (I.J.D.)

**Keywords:** magnetic resonance, linear accelerator, MRgRT, radiotherapy, image-guided radiotherapy, IGRT, adaptive therapy

## Abstract

Advances in image-guided radiotherapy have brought about improved oncologic outcomes and reduced toxicity. The next generation of image guidance in the form of magnetic resonance imaging (MRI) will improve visualization of tumors and make radiation treatment adaptation possible. In this review, we discuss the role that MRI plays in radiotherapy, with a focus on the integration of MRI with the linear accelerator. The MR linear accelerator (MR-Linac) will provide real-time imaging, help assess motion management, and provide online adaptive therapy. Potential advantages and the current state of these MR-Linacs are highlighted, with a discussion of six different clinical scenarios, leading into a discussion on the future role of these machines in clinical workflows.

## 1. The Clinical Role of the MR-Linac

The introduction of magnetic resonance-guided radiotherapy (MRgRT) in clinical practice has addressed multiple problems associated with traditional computer tomography-guided radiotherapy (CTgRT). MRgRT has been validated in multiple settings as not only feasible, but efficacious with either comparable or improved clinical outcomes [1,2,3,4]. Its applications are on the rise in multiple locally advanced and metastatic sites [5].

The current standard of image-guided radiotherapy (IGRT) utilizes CT scans for initial treatment planning and relies on kilovoltage (kV) static images or cone beam CT’s (CBCT) for setup verification, termed “pre-treatment imaging”. CTgRT modalities come with many inherent issues that limit the precision in radiotherapy treatment planning and delivery. For instance, CT imaging relies on differences in attenuation properties and electron densities of tissues to delineate structures [6]. This is very difficult in the abdomen, where each organ is of similar electron density and in close proximity to one another. The current paradigm of radiotherapy also relies on utilizing the original plan for daily treatments across multiple weeks without significant alteration. However, highly conformal treatments require setup reproducibility and accommodation for changes in the position of the patient or internal organs. Issues commonly faced in the clinic are those of intra-fractional and inter-fractional changes of anatomical structure—the variation in position observed within and between treatments, respectively. With most treatment modalities, a patient’s daily alignment can only be adjusted in small increments by shifting the treatment couch. There are currently very few options for adapting treatment plans based on anatomical or physiological change. If treatment alignment is felt to be suboptimal, patients must stop their course of radiotherapy and undergo a long process of replanning. This can be arduous and time intensive, delaying therapy.

Intra-fractional movement of organs in dynamic regions of the body is reduced by treatment setup devices. Motion is typically accounted for during treatment planning by use of geometric expansions or four-dimensional CT (4DCT) scans across time. Both methods produce target volumes that inherently sacrifice normal tissue to ensure target coverage during the entire cycles of motion. An additional method of accounting for motion is beam gating. Gated treatments allow the dose delivery only at optimal instances. However, current methods of gating track motion by the use of an external camera or internal fiducial markers, both of which rely on anatomic correlates that may not provide accurate targeting [7].

Recently popularized MRgRT workflows overcome the drawbacks of CTgRT and have been bolstered by the implementation of the MR-Linac. Daily MRI acquired by these systems facilitates improved soft tissue delineation. Apart from better visualization of organs at risk (OARs), the devices can review original dose distribution on each fractional scan and offer the ability to adapt the original plan to accommodate for positional changes. The intent of adapting the original plan provides better target coverage and safer administration of radiation by limiting the dose to OARs. This is critical in areas of the body with variable filling of luminal organs, such as the rectum, large intestine, and bladder, which contribute to setup incongruence and change of internal anatomy [8].

In contrast to conventional linear accelerators, MR-Linac also offers the ability to produce real-time images during the treatment delivery. Real-time imaging allows for live MRI visualization of the target and treatment gating based on tumor position. Dose is administered only while the tumor lies within the gating window. This technique improves confidence in the target position. Consequently, a smaller expansion can be made on the gross tumor volume (GTV) and minimize overlap of target volumes with OARs. Effectively, reducing margins will limit the volumetric dose received by the normal tissue, which could translate to reduced toxicity or increased quality of life. The principle of gating is critical in regions associated with frequent motion, such as the region surrounding the diaphragm.

The overall purpose of any advance in radiotherapy is to either optimize dose delivery to the target or minimize the dose to OARs, with the overall goal of raising the therapeutic index. This process increases the tumor control probability and reduces the normal tissue complication probability. In the following discussion, each of these benefits will be exemplified by clinical situations, highlighting the advantages of MRgRT as well as MR-Linacs over traditional CTgRT treatment. Trials and retrospective analyses on this new technology are limited at this early stage of adaptation. While experience with these systems is still in its infancy, treatment extends beyond the following applications and will continue to burgeon in the coming years.

## 2. Pancreatic Cancer

Pancreatic cancer is often associated with a challenging early diagnosis and a high mortality rate. The only curative option for this disease is surgical resection, yet only about 15% to 20% of patients are diagnosed at a time where the disease remains in a resectable state [9,10]. Cases where the malignancy invades nearby blood vessels or other organs are defined as unresectable depending on the level of involvement, but can be classified as borderline resectable if there is relatively less involvement. Radiotherapy and chemotherapy play a role in the management of disease by serving as definitive modalities in unresectable cases, a method of symptom palliation, or as a neoadjuvant option with the objective of getting borderline resectable patients to a resectable state of disease by cytoreduction [9,10]. More recently, delivery of definitive radiotherapy in unresectable situations has been shifting toward stereotactic body radiotherapy (SBRT), which delivers a higher dose in fewer treatments (i.e., hypofractionation) compared to standard fractionation regimens. This reduces the time to initiation of chemotherapy and the overall treatment duration [11].

An obstacle in the incorporation of SBRT into treatment of this disease is avoiding the OARs around the pancreas. The head of the pancreas sits immediately adjacent to the duodenum and is directly associated with the common bile duct, while the neck, body, and tail of the pancreas may lie near the jejunum, stomach, kidneys, spleen, and several major blood vessels. High doses to these structures can cause bowel or gastric ulcers, biliary stricture, or renal dysfunction [12,13]. Treatment with radiotherapy must consider the tolerance dose for these structures during planning and delivery to avoid significant toxicity.

The safety of such ultra-hypofractionated treatments was observed in a retrospective study from Stanford University, in which patients with unresectable pancreatic adenocarcinoma were treated with SBRT consisting of 25 Gy in 1 fraction [12]. Grade 2 and higher toxicities were observed in 17% and 28% of patients, respectively. The most common adverse effects were ulcers of either the small bowel or stomach, which ultimately represented significant morbidity for these patients.

A subsequent retrospective review from the same group at Stanford compared single-fraction SBRT to multi-fraction SBRT [13]. The review showed reduced toxicity in the multi-fraction arm when compared to the single-fraction arm while maintaining similar oncologic outcomes. Local recurrence by 12 months was found to be 9.5% in the single-fraction arm and 11.7% in the multi-fraction arm, without statistical significance in the difference. Meanwhile, cumulative gastrointestinal toxicity of grade 3 or higher by 12 months was 12.3% in the single-fraction arm and 5.6% in the multi-fraction arm [13]. Current SBRT regimens in this setting typically range from 3 to 5 fractions to reflect these data. Despite improvements, morbidity from radiotherapy in unresectable pancreatic cancer may require medical intervention and result in a treatment delay, which may further delay the initiation of chemotherapy, reduce the efficacy of treatment, and lose the chance of attaining a resectable state for definitive therapy.

**Figure 1:**
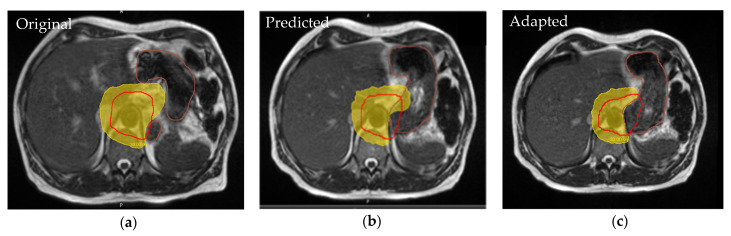
The 30 Gy (yellow) isodose lines are displayed for original, predicted, and adapted plans, with PTV in red and stomach in brown. Clinically set constraint (0.03 cc < 30 Gy) achieved by the adapted plan (**c**) was comparable to the original plan (**a**), however the dose constraint for the predicted dose (**b**) was not met due to changes in stomach volume and positioning.

Treatment-associated morbidity could in part be caused by the inability to accurately visualize the tumor during treatment due to poor soft tissue delineation, which ultimately requires larger volumetric expansions to cover the uncertainty and increases the dose to OARs. Toxicity can be reduced by limiting the radiation dose received by OARs. While initial treatment planning may meet dosimetric constraints, the current standard of CTgRT does not adapt daily treatments beyond minimal geometric shifts of the treatment table. Thus, significant variations from the initial setup are not properly assessed during treatment. Using the onboard MR imaging capabilities of MR-Linacs, changes in treatment setup or anatomy can be accounted for in adaptive planning. During this process, daily fractional scans are used by the treating physician and physicist to adjust the radiotherapy plan to not only optimize the target coverage, but also avoid critical adjacent OARs [5,14,15]. This can be observed in Figure 1, where the initial volumes for treatment are modified based on daily MRI to avoid the spillage of the 30 Gy isodose line, in yellow, into the stomach, in brown.

A study from Italy investigated the potential dosimetric improvement using an online adaptive workflow on an MR-Linac in patients with locally advanced pancreatic cancer [14]. A total of 8 patients were planned to receive 5 fractions of SBRT with total dose between 30 and 40 Gy. Out of 38 total treatments delivered, 26 were adapted at the time of treatment, equal to 68.4% of treatments. This led to improved dosimetry, and plan adaptation increased the planning target volume coverage (PTV) by 10.8% and showed an overall trend in reducing the dose received by the duodenum and stomach. At a median follow-up of 13 months, only one grade 1 toxicity was reported with no late toxicity, which is greatly reduced when compared to historic standards [12,13]. Results from this study suggest that a majority of treatments by standard CTgRT with daily CBCT would require adapting if better imaging was available during the time of treatment. This represents under-coverage of target volume and overdosing the OARs with conventional treatments.

Further evidence of benefit is provided by a multi-institutional retrospective review of patients with inoperable pancreatic cancer that included 44 patients [16]. The review compared different radiotherapy regimens of either conventional fractionation, hypofractionation, or SBRT. Those who received 15 or fewer fractions (i.e., hypofractionated or SBRT regimens) were treated with adaptive MRgRT. At median follow-up of 17 months, those who were treated with high-dose SBRT on the MRgRT workflow had a statistically significant increase in 2-year overall survival when compared to the rest of the cohort. Additionally, grade 3 or higher toxicity was not observed in this MRgRT subgroup, while 3 patients experienced this outcome in the standard dose subgroup treated with traditional CTgRT workflow. Similar data have been reported from a similar retrospective, single-institution study of 35 patients with locally advanced pancreatic cancer treated with MR-guided SBRT [17]. Use of adaptive therapy was able to minimize toxicity and provide excellent oncologic outcomes.

Finally, a prospective phase I trial utilizing SBRT and online adaptive radiotherapy for abdominal metastases or primary disease has shown good dosimetry with minimal toxicity [1]. The trial included 20 patients, 2 of which had primary, unresectable pancreatic disease undergoing ablative SBRT. Any adapted treatments were subjected to hard OAR constraints with online adapted plans created for 81 of 97 total fractions delivered, 61 of which were due to an OAR constraint violation. After a median six-month follow-up, there were no acute grade 3 or higher toxicity events observed. Similarly, promising outcomes were also reported in a single institutional study of 44 patients with inoperable pancreatic cancer treated using SBRT with adaptive planning by MR-Linac [18].

Overall, these findings suggest that real-time adaptation of radiotherapy plans using MRgRT in the setting of pancreatic cancer can reduce toxicity by providing better dosimetry from adaptive planning. This has the potential to reduce the overall duration of therapy by prohibiting delays to chemotherapy, improving outcomes, and reducing the burden of morbidity while obtaining a resectable state.

## 3. Thoracic Malignancies

One of the greatest challenges in treating malignancies of the thoracic cavity is accounting for motion during treatment. There are several approaches to managing intra-fractional motion. Utilization of commercially available or in-house-developed site-specific devices for treatment setup is one popular approach. These include abdominal compression belts that limit diaphragmatic exertion, applying methods of respiratory gating, or accounting for total motion of the target using 4DCT across the respiratory cycle [19]. Ultimately, such techniques account for motion by either limiting the change in position of the target or allowing the planning physician to visualize the location of the tumor across an entire breathing cycle. During planning, the PTV is typically generated by expanding the internal target volumes (ITV) contoured on 4DCT scans. This PTV is typically not altered through the course of radiotherapy, with only minor geometric shifts in the treatment table used to align the patient.

None of these systems provide real-time visualization of the tumor during treatment delivery and there is no mechanism of tracking the anatomical variations between and within treatments, consequently raising the probability of a geographic miss from the original plan. Such discrepancies in treatment delivery are far more detrimental for hypofractionated or ultra-hypofractionated SBRT treatments, where precision is a prerequisite, and any missed dose per fraction could account for a larger portion of the total dose.

A clinical example where target tracking is pertinent is the treatment of peripheral lung malignancies. When located in the lower or middle lobes, these tumors are under significant influence from intra-fractional diaphragmatic motion [20]. The current standard for early-stage peripheral non-small cell lung cancers (NSCLC) is either definitive surgical resection or ablative radiotherapy if the patient is deemed inoperable [21]. Effective SBRT in this setting requires delivery of an over 100 Gy biological equivalent dose in 5 or fewer fractions, resulting in large quantities of the dose delivered per treatment [22]. Use of SBRT with the real-time gating capabilities of MR-Linacs allows for potential improvement in this realm. Real-time tracking of the structure of interest can offer greater confidence in the localization and irradiation of the tumor compared to standard techniques of geometric expansion. Higher confidence will ultimately translate to more conservative target volume expansion, and a reduced dose delivered to normal lungs.

This theoretical improvement has been observed in dosimetric studies. One study of 23 patients with 25 early-stage peripheral NSCLC lesions underwent a total of 128 fractions of MR-guided SBRT [23]. A 4DCT was also used for treatment planning in a subgroup of 14 patients. On comparison amongst patients with both imaging modalities, the study found that planning target volumes created using gating and adapting on MR-Linacs were 53.7% smaller than those created on the conventional 4DCT. Thus, improved localization during treatment facilitated by MRgRT has the potential to ultimately reduce the total dose delivered to the normal lung tissue in proximity.

Furthermore, the same study [23] compared 112 plans that were adapted based on daily MR imaging to their corresponding predicted plans created at the original simulation. The authors found that on-table adaptation improved PTV coverage in 58% of cases by raising the median dose to the target or increasing the portion of the target covered by the prescribed dose. In this study, there is dosimetric evidence that MRgRT cannot only reduce the dose to normal structures but also increase the accuracy when compared to standard CTgRT.

Discussion to this point has regarded peripheral lung tumors, distinct from central or ultra-central lesions. Multiple sensitive OARs including the heart, bronchi, esophagus, and spinal cord lie within the central thoracic cavity. High doses associated with ablative SBRT in treatment of more centrally located NSCLC can lead to morbid or fatal outcomes due to the adjacency of such structures. Reported adverse events have included hemorrhage, bronchial collapse, acute esophagitis, and more [24]. Incidence of these outcomes is increased when treating “central” lung tumors, those in which the PTV is abutting or falling within 2 cm of the proximal bronchial tree or trachea [25]. Chances are further increased in treating “ultra-central” tumors with ablative doses, or those which have a gross tumor directly contacting the central structures [26].

To deliver safe radiotherapy, the dosimetric constraints of adjacent thoracic organs are often the primary dose-limiting factor for safe and optimal radiotherapy treatment. To examine the benefit of MRgRT in this space, a retrospective study of 25 patients with central lung tumors who were treated with SBRT delivered by MR-Linac using the gated breath hold technique was conducted [27]. A total of 182 fractions of SBRT were delivered in this cohort, of which 168 plans were adapted and allowed for direct comparison to their original, predicted plans. There was a significant decrease in OAR constraint violations from the predicted plans to the adapted plans (127 versus 93, respectively). Longer-term follow-up will reveal if these observed dosimetric benefits correlate to decreased toxicity.

Early clinical data are already available in this realm. In a very similar phase I trial of ultra-central lung tumors, over 70% of plans were adapted for the purpose of OAR constraint reversal [3]. Follow-up of the five enrolled patients at six months showed 100% local control with no grade 3 or higher acute adverse events—a great departure from earlier trials with up to 54% of patients experiencing severe toxicity at long-term follow-up [25].

Overall, treatment of both primary and oligometastatic lesions in the thorax to ablative doses is complicated by the adjacency to sensitive OARs and unpredictable intra-fractional motion [28]. The thorax is a dynamic environment of a beating vascular system, a constricting esophagus, and a bellowing diaphragm. Standard CTgRT treatments create rigid plans consisting of volumetric expansions on predicted tumor motion to account for these confounders, with limited daily visualization. In doing so, more normal tissue receives radiation, and geographic misses can lead to reduced target coverage or damage to OARs. The implementation of MRgRT with adapted plans and gated delivery on MR-Linacs has improved dosimetry across multiple examples, and early trial data have shown correlation to improved clinical outcomes.

## 4. Prostatic Malignancies

Radiotherapy plays a significant role in the management of prostate cancer. One of the newer roles it has taken on is the fast and definitive treatment of localized prostate cancers through the use of SBRT [29]. Typically, a large portion of these patients would also be candidates for active surveillance, but many patients prefer a more curative approach to the disease with avoidance of surgical intervention.

SBRT for localized prostate cancer has been proven effective by many trials [30,31,32], but most trials only have short-term follow-up data available at this time. A study of 515 patients with localized prostate cancer of mostly low- and intermediate-risk disease were prospectively followed after receiving SBRT [32]. At median follow-up of 84 months, the 7-year disease-free survival (DFS) was comparable between the favorable intermediate- and low-risk populations, with 95.2% and 93.2%, respectively. However, there was a sharp falloff of DFS in the unfavorable intermediate-risk population, with only 68.2% at 7 years. These results have led to the widespread adoption of SBRT in only low- and favorable intermediate-risk disease, while those who are unfavorable intermediate-risk are often recommended a more conventional fractionation [32].

Acute side effects from external beam radiotherapy in prostate cancer typically include dysuria, urinary frequency, diarrhea, and rectal urgency. Long-term side effects can include urethral stricture, cystitis, proctitis, and sexual dysfunction [31]. Therefore, toxicity is of great concern with the use of SBRT in sensitive areas surrounded by the urethra, bladder, rectum, and bowel. The connecting nerves of the prostatic plexus responsible for proper sexual function are also at risk.

**Figure 2:**
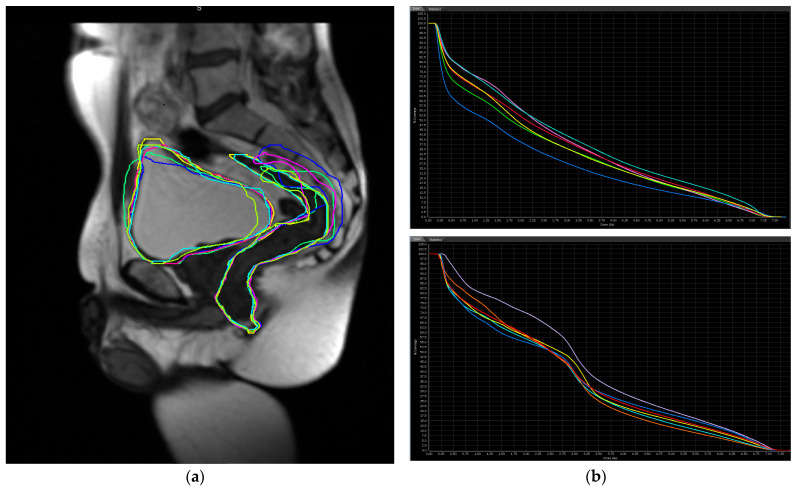
(**a**) Sagittal MR view of the pelvis with delineation of bowel and bladder volumes across multiple treatments using an MR-guided linear accelerator. (**b**) Dose volume histograms showing variability in received dose to the bladder (top) and rectum (bottom) across daily treatments depending on position and indicated by different line colors.

With the implementation of MR-Linacs across multiple institutions, the question has been posed if these observed toxicities can be reduced by assessing inter-fractional motion with daily MRI. The ability to adapt treatments to changes in structures between fractions holds great therapeutic potential in the prostate due to the dynamic motion and filling of the rectum, bowel, and bladder. This is exemplified by Figure 2, which shows multiple iterations of bowel and rectal contours across treatments that are overlayed to depict the variation that can be observed. The changes in dose due to alteration of internal anatomy on CTgRT have been elaborated and quantified, however such approaches are impractical, adding a significant amount of manpower [33,34].

This theoretical benefit was observed clinically in a prospective, single-arm phase II trial of 101 patients with mostly intermediate- or high-risk localized prostate cancer treated on an MR-Linac with SBRT [4]. The trial showed that maximum acute genitourinary toxicity of grade 2 or higher peaked at 23.8%, which is much less than that observed in larger phase III trials of moderate hypofractionation with a similar high-risk patient population who underwent planning with standard CTgRT [35,36]. They also observed a very low rate of early grade 2 gastrointestinal toxicity, measuring 5% incidence with no incidence of grade 3 toxicity [4]. These early trial data suggest a clinical benefit to the use of MRgRT for definitive SBRT in the treatment of low- to favorable intermediate-risk prostate cancer.

Further investigation in this area is underway. A phase III randomized trial performed at a single center is currently comparing outcomes of MR-guided SBRT for intact prostate cancer to CT-guided therapy, with the primary endpoint being acute grade 2 or higher toxicity [37]. Interim analysis shows a significant reduction in acute genitourinary toxicity with MR guidance over CT guidance, with improved urinary and bowel function [37]. Final analysis of the patient cohort will help to clarify the role of MRgRT in the setting of intact, localized prostate cancer.

In summary, the high prevalence of prostatic adenocarcinoma demands safe and efficacious treatment with minimal burden of toxicity. Use of SBRT has become widely adopted due to its proven oncologic equivalence to protracted regimens, and acceptable levels of toxicity. Application of the changes observed on daily MRI and the ability for adaptive treatments provided by an MR-Linac may stand to further reduce the toxicity experienced in this population by accounting for changes in rectal, bowel, and bladder positioning. This benefit could translate to other organs in the abdomen or pelvis as well [38,39].

## 5. Intrahepatic Malignancies

**Figure 3:**
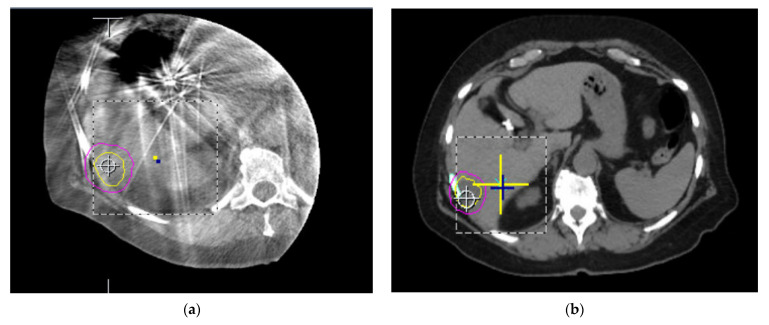

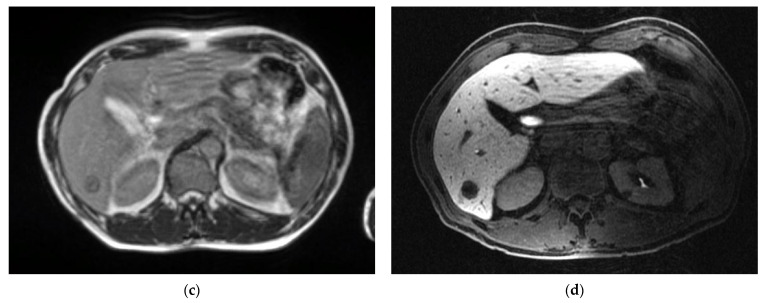
Comparison of different imaging modalities for the soft tissues of the abdomen. (**a**) Axial CBCT imaging of the liver taken on a conventional linear accelerator. (**b**) Axial CT slice of the abdomen used for planning with each organ of a similar electron density. (**c**) Axial slice of the abdomen acquired with 0.35 T MRI. (**d**) Axial slice of the abdomen using 1.5 T MRI. Lesion identification is much easier on both 0.35 and 1.5 T MRI images compared to CT and CBCT scans.

As discussed, treatment of abdominal malignancies is complicated by the proximity of critical structures, movement of the diaphragm during treatment, and change in organ volume between treatments. The similarity in electron density amongst all tissues within the cavity further complicates the planning and delivery of radiotherapy using traditional CTgRT. This is exemplified by the poor visualization of hepatic or bile duct disease on standard imaging. Daily localization in the current paradigm utilizes kilovoltage X-ray imaging, CBCT, megavoltage CT, or CT on rails, all of which ultimately rely on differences in electron density for discrimination between nearby organs. The result is very poor soft tissue contrast, making verification and adaptation of treatment difficult [15,40,41]. Figure 3 shows the wide range of imaging available clinically and the resulting ability to depict different soft tissues. Localization can also be aided by administering IV contrast or fusion of diagnostic scans. However, any image fusion is subject to variability between the operator and changes in anatomy or positioning [42].

This issue becomes evident when attempting to visualize lesions within the liver. Not only is it difficult to delineate the intrahepatic target itself, but it is often difficult to delineate OARs such as the connected duodenum and intrahepatic bile duct. In this situation, MR imaging provides a superior ability to discern soft tissue structures [43]. For example, a quantitative contouring study of abdominal patients with both onboard MR compared to standard CBCT imaging showed that MRI resulted in better visualization for 77% of abdominal target and OARs [40]. The study also observed improved agreement between physician contours with onboard MR delineation when compared to agreement on CBCT delineation.

Poor visualization is partially ameliorated by the use of electron-dense fiducials placed in or near soft tissue that are easily visible on kilovoltage imaging [44]. However, there are risks associated with such approach. Procedural complications associated with implantation of the markers have been estimated at around 5% and 17.3% for major and minor complications, respectively, across multiple organ implantations [45]. Migration of markers after successful placement is also a potential confounder during radiotherapy planning. Local migration has been reported in about 2–5% of cases [45,46]. If it occurs between simulation and treatment, it poses a risk of geographic miss of the target if relying heavily on their anatomic positioning.

The same risk is posed to OARs, which are often freely mobile within the peritoneal cavity. While dosimetric constraints can be met on initial planning, movement between or within treatments may occur that would violate these constraints if re-planned. The position of OARs may encroach upon the target with little adjustment of the radiotherapy plan, made possible with the current CT-derived standard. Thus, better visualization is yet another avenue in which the MRgRT has the potential to improve on the current standard of radiotherapy. Using onboard MRI, the target can be more precisely defined, and the positioning of OARs can be accurately assessed on a per-treatment basis. From these, a new plan can be devised if target coverage or dosimetric constraints to nearby organs are not met.

The need for precision in dose delivery is exemplified by intrahepatic cholangiocarcinoma, where targets are within the liver and can be adjacent to many structures at risk. Surgical resection with negative margins is the only curative modality for cholangiocarcinoma, yet most patients are deemed to be unresectable at presentation due to local extension [47]. Those who are not deemed to be surgical candidates undergo chemoradiation with consideration of transplantation. In such settings, radiotherapy serves as the local control mechanism. A retrospective analysis showed that reaching a biological effective dose (BED) over 80.5 Gy is associated with long-term survival benefits in localized, inoperable intrahepatic cholangiocarcinoma [48]. The retrospective study observed 3-year overall survival of 78% in patients who received BED over 80.5 Gy versus 38% 3-year overall survival in those who did not. These oncologic outcomes are comparable to those who have operable intrahepatic cholangiocarcinoma and undergo surgical resection. The remaining limit on administering these high doses is the constraints of adjacent OARs.

With such high doses of radiation, toxicity becomes a greater concern. A series of 17 patients with unresectable, locally advanced cholangiocarcinoma were treated with SBRT using MRgRT at a single institution [49]. They observed only one patient with acute grade 3 gastrointestinal toxicity, representing 6% of patients, a large improvement when compared to historic standards of 10% to 26% experiencing grade ≥ 3 GI toxicity on meta-analysis [50]. It is likely that the reduced toxicity resulted from improved visualization and the ability to adapt radiotherapy plans that is afforded by MRgRT.

Similar principles apply for primary or metastatic lesions of the liver itself. Dosimetric benefits of the MRgRT have been described in these cases, including better PTV coverage and sparing of the bowel and duodenum [51,52]. Ablative doses have also been delivered safely and effectively to intrahepatic targets using MRgRT in a multi-institutional series [2]. In an aforementioned prospective phase I trial of patients with intrabdominal malignancy, use of MR-guided SBRT in patients with intrahepatic disease was deemed technically feasible and safe [1].

Intrahepatic disease serves as a great example of the strength of MR imaging in combination with radiotherapy. The improved soft tissue differentiation can help to identify malignancy while avoiding normal structures to provide more efficacious definitive treatment and avoid the need for placement of fiducials. Furthermore, it can ensure accurate delivery of high doses of radiation via SBRT techniques that can improve oncologic outcomes.

## 6. Pediatric Malignancies

When treating pediatric patients with radiotherapy, physicians must be a strict steward of dose distribution. Every exposure is impactful, as any unwarranted dose can compound the patient’s risk of secondary malignancy in their potentially long life after treatment of their disease [53,54]. For this reason, diagnostic scans that use ionizing radiation are generally limited as much as possible in this young population, and all attempts at therapeutic dose limiting are taken (e.g., consideration of proton therapy).

During a typical course of radiotherapy, the patient is put through many imaging modalities outside of the initial workup and diagnosis. Simulation CT scans for radiotherapy planning, daily imaging in the form of kilovoltage X-rays, and MVCT or CBCT for setup verification are all sources of non-therapeutic ionizing radiation. Although the dose received from imaging modalities is minimal compared to therapeutic radiotherapy doses, the risk of secondary malignancy from them can still be estimated and avoided [55,56].

**Figure 4:**
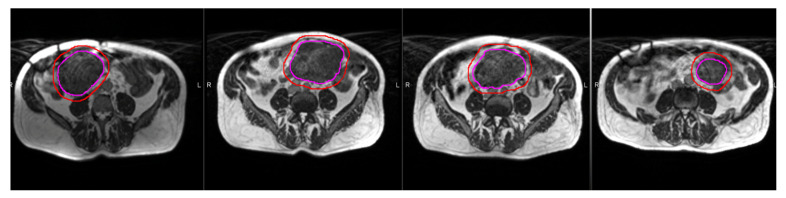
Treatment response assessment on serial MR images during a course of radiotherapy, with images moving chronologically from left to right. Note the visual reduction in gross tumor volume (pink) and overall reduction subsequently expanded target volume (red), as well as the freely mobile target in the abdominal cavity across multiple weeks.

Pediatric patients can stand to benefit from MRgRT in multiple ways: by reducing the non-therapeutic dose from imaging and by reducing the total therapeutic dose delivered to the patient. MRI is a non-ionizing form of imaging that provides excellent soft tissue visualization for contour delineation and can serve to replace the current standard of CTgRT. Beyond imaging, this population also benefits from the improved targeting associated with MR incorporation into the linear accelerator. As previously mentioned, when confidence in the target is increased, the traditional marginal expansions on target volumes can be reduced [23]. One example is the use of gated treatment based on live sagittal MR images afforded by MR-Linacs. Gating based on live imaging can be utilized in targets with a large component of motion to reduce volumetric expansion and expose less normal tissue during each treatment. The importance of this practice is compounded in a young population that cannot actively participate in gating techniques such as inspiratory or expiratory breath hold. Finally, treatment response during the course of radiotherapy can be assessed and open the possibility of adapting plans for an outright target volume reduction by replanning. An example of volume reduction afforded by treatment response is shown in Figure 4. Treatment response can be assessed by subtle changes in imaging and could potentially serve as a prognostic indicator [57,58]. While not clinically validated in this population, the use of imaging biomarkers could help guide treatment escalation or de-escalation in the future and avoid any excess radiotherapy in pediatric patients [41].

These factors come together to reduce the normal tissue exposed and total dose delivered during radiotherapy, which can be of exponential benefit in pediatric patients. In the first case report of pediatric treatment with a MR-Linac, a 3-year-old underwent treatment radiotherapy for stage III thoracic rhabdomyosarcoma [59]. The group describes reduced marginal CTV to PTV expansion from the typical 1.0 cm to a tighter 0.5 cm with the use of MR gating. They estimated a 59% reduction in PTV volumes from this adjustment, sparing a large volume of normal tissue [59].

A second case report in the treatment of a 1.5-year-old with extracranial malignant rhabdoid tumor with metastatic lesion in the liver showed good tolerance of SBRT with a 40.8% volumetric reduction of tumor by using MRgRT [60]. All fractions underwent an adaptive approach with the application of the predicted plan onto daily MR imaging and adaptation of volumes if OAR constraints were not met. Three of five fractions required re-planning for this reason, suggesting that non-ideal dosimetry may be occurring in most conventional treatments on CTgRT. Widening the therapeutic index in this population has relatively more impact when compared to older patients who may not experience more long-term benefit or morbidity of radiotherapy.

Although these benefits are theoretically sound, there are multiple concerns that arise when treating pediatric patients via adaptation on a MR-Linac. This technique often requires extensive time for volume delineation, replanning, and assessment of new dosimetry, translating to prolonged time on the treatment couch, which may not be feasible in a young population [15]. Secondly, pediatric patients often have difficulty tolerating long procedures and will require use of anesthesia [59,60]. The equipment required for anesthesia must be made to be MR-compatible, and the vault that houses the linear accelerator must be accommodating to these often-bulky machines. Treatment times in most studies of adults have consistently shown a duration ranging from 50 to 80 min with the use of adaptation [1,3,27,51,61], the impact of which is compounded in the setting of anesthesia use. Finally, the effect of the magnetic field must be considered when calculating dose deposition during planning, as secondary electron paths may be influenced by the magnetic field of the MR-Linac [62,63]. While this effect is worrisome when treating patients of any age, errant dose deposition can be more impactful in the pediatric setting due to the prolonged risk of secondary malignancy.

To describe the effects of the electron path in the presence of a magnetic field, a study from The Netherlands compared the magnitude of the electron return effect in various magnetic field strengths and electron energies. They found minimal effect on the dose distribution in soft tissue or air cavities at therapeutic doses in lower field magnets (0.2 T in this study) due to the large rotational path of secondary electrons [63]. However, at higher field strengths (up to 3.0 T in this study), there was a significant change in the lateral dosing due to the electron return effect [63]. In the aforementioned case report, a separate team found that electrons exiting soft tissue on the distal end of the beam contribute a small percentage of the dose to the periphery, comparable to a typical skin dose and not clinically impactful [59]. While easily manageable, confounders such as the Lorentz effect must be kept in mind to avoid adverse outcomes and are more substantial when working under higher magnetic field strengths.

Pediatric malignancies represent a situation in which the patient benefits from multiple advances provided by the MRgRT, with the culmination in the greater benefit of overall dose reduction. While this is given greater priority in the pediatric population due to their increased time to develop toxicity or secondary malignancy, it could become the standard of radiotherapy for all disease sites and ages as we aspire to expand the therapeutic index for all patients.

## 7. Future Endeavors

Treatments with MRgRT and MR-Linacs are showing improved dosimetry in multiple anatomic sites and applications, with early trials showing reduced toxicity with equivalent outcomes to CT-based radiotherapy [1,2,3,4,37,64]. MR-capable radiotherapy provides the ability for adaptive plans without significant deviation from the standard clinical workflow. Replanning can help avoid geometric misses by accounting for inter-fractional change. Real-time MRI and gated radiotherapy allow for tighter marginal expansions and treatment in more sensitive areas of the body. In combination with smaller margins, better visualization of soft tissues can help avoid sensitive structures and OARs. Future work regarding treatments using MRgRT workflows will entail the perfection of the technique, a reduction of treatment time, and continued progression of these unique abilities to improve oncologic outcomes.

One such endeavor is taking the field of radiotherapy to where it was once forbidden—treatment of intracardiac targets. Usually considered an OAR to be avoided, the heart represents an area of the body that is usually off limits for radiotherapy penetration. However, recent evidence has shown that radiotherapy can help treat both benign and malignant diseases involving the heart by using newer technology and improved imaging [65].

Patients with ventricular tachycardia (VT) suffer from an irregular electrical circuitry of the myocardium that can be fatal if left untreated. Typical management of VT first includes antiarrhythmic medications [66]. If these are insufficient, the patient typically undergoes catheter ablation of the irregular myocardium. However, ablation is often aborted or will have acute failure in about 10% of cases due to the sensitive location of abhorrent myocardium or the inaccessibility of the tissue [67]. On top of the risk of failure of the procedure, an estimated 5% of patients undergoing radiofrequency ablation will experience early post-procedural mortality [68]. In this niche lies the potential for improvement by applying new techniques of external beam radiotherapy in the form of ablative SBRT.

Animal models testing this idea showed that a dose range of 25 to 35 Gy in a single-fraction SBRT were sufficient to induce fibrosis and render the treated cardiac tissue inert [69,70]. Other case series had shown feasibility of such procedures in humans [71]. Based on these findings, a small group of five patients with refractory VT who had contraindications to cardiac ablation or had failed at least one ablation were enrolled to undergo SBRT to the sites of the aberrant signal [72]. Each patient was treated to 25 Gy in a single fraction using conventional SBRT techniques on the CT-based planning scan, with additional cardiac information gained from electrocardiographic mapping and cardiac MRI when available. Results were significant and outstanding—a reduction of 99.9% was observed in VT episodes over the nearly 4 years after the initial 6-week blanking period following ablative procedures [72]. These data established the new boundaries of what can be performed with SBRT. A subsequent phase I/II trial from the same group that observed outcomes of noninvasive radio-ablation proved to be moderately safe. Follow-up at 90 days after the completion of treatment showed that 10.5% of patients experienced a serious treatment-related adverse event with no acute toxicity observed after SBRT [65]. This represents a safe alternative for patients who cannot undergo radio-ablation and may become a first-line option with further investigation.

An additional source of intracardiac target is the rare case of malignant involvement. Primary tumors of the heart are very rare, found in about 0.02% to 0.06% in large autopsy series [73,74]. However, secondary involvement from metastatic disease or local progression is more common and estimated to be around 8.4% in patients who have died of neoplastic process, with the most common primary disease being metastatic melanoma and locally advanced lung cancers [75,76]. As systemic therapies advance, patients live long enough to develop metastases of multiple organs, and cardiac involvement has become more prevalent in recent trials [77]. Furthermore, patients with a low metastatic burden can undergo salvage or curative radiotherapy with increasing success [78,79,80]. Given the success of treating benign intracardiac disease and the improved precision associated with MRgRT, the next logical step is treating malignant targets within the heart.

One group successfully treated five patients with cardiac or pericardial metastatic lesions who were symptomatic from involvement by using MRgRT [81]. The median prescribed dose to PTV was 40 Gy, with a range of 40–50 Gy delivered in 5 fractions on non-consecutive days. The group utilized multiple treatment setups and used the real-time sagittal MR images to gate the tumor with a uniform PTV expansion of only 3 mm from GTV. Of the five patients, one experienced complete response, two experienced partial response, and the other two experienced stable disease after a median follow-up of 1.5 months. All patients had relief of some symptoms, with one patient developing worse dyspnea and two experiencing mild fatigue. Overall, the group concluded that MRgRT provides the ability to successfully treat these intracardiac metastases with no serious adverse events.

In summary, magnetic resonance-guided radiotherapy has addressed many areas of uncertainty associated with the current standard of CT-based radiotherapy. Benefits include improved soft tissue delineation, safer treatment of sensitive anatomic sites, reduced toxicity observed in trials, simple motion management, real-time gating of targets, and an overall reduced dose to patients. Using this new paradigm, boundaries will continue to be expanded in radiotherapy to provide more efficacious treatments to a population of patients who desperately need it.

## Data Availability

Not applicable.
